# Planning Skills in Autism Spectrum Disorder Across the Lifespan: A Meta-analysis and Meta-regression

**DOI:** 10.1007/s10803-016-3013-0

**Published:** 2017-02-03

**Authors:** Linda M. E. Olde Dubbelink, Hilde M. Geurts

**Affiliations:** 1 Dr. Leo Kannerhuis, Houtsniplaan 1, 6865 XZ Doowerth, The Netherlands; 2 Dutch Autism & ADHD Research Center (d’Arc), Department of Psychology, Division Brain & Cognition, University of Amsterdam, Nieuwe Achtergracht 129-B, 1018 WS Amsterdam, The Netherlands

**Keywords:** ASD, Planning, Meta-analysis, Age, Task-type, IQ

## Abstract

Individuals with an autism spectrum disorder (ASD) are thought to encounter planning difficulties, but experimental research regarding the mastery of planning in ASD is inconsistent. By means of a meta-analysis of 50 planning studies with a combined sample size of 1755 individuals with and 1642 without ASD, we aim to determine whether planning difficulties do exist and which factors contribute to this. Planning problems were evident in individuals with ASD (Hedges’*g* = 0.52), even when taking publication bias into account (Hedges’*g* = 0.37). Neither age, nor task-type, nor IQ reduced the observed heterogeneity, suggesting that these were not crucial moderators within the current meta-analysis. However, while we showed that ASD individuals encounter planning difficulties, the bias towards publishing positive findings restricts strong conclusions regarding the role of potential moderators.

## Introduction

Planning is defined as choosing and implementing a strategy in new or routine situations in which a sequence of planned actions must be monitored, judged and updated in light of a pre-specified goal (Hill 2004; Ward and Morris 2005). This complex cognitive ability enables us to perform adaptive behavior. Whether we make to do lists, schedule appointments, organize our social life, or write an article, planning both directs and evaluates our behavior.

People with autism spectrum disorders (ASD) are thought to encounter planning difficulties (e.g. Hill 2004; Lopez et al. 2005; Van den Bergh et al. 2014). They have trouble organizing their daily life, maintaining (social) activities or coping with unregulated stretches of time (APA 2013; Ozonoff et al. 2002). Reports of caregivers also indicate planning deficits in the daily life of their child in comparison to their typically developing peers (Rosenthal et al. 2013; Van den Bergh et al. 2014). Reviewing research on planning performance on cognitive measures in ASD yields, however, inconsistent findings, resulting in a lack of clarity on the mastery of this skill in ASD. Some studies do not observe differences in terms of planning performance between people with ASD and typically developing individuals (e.g. Bölte et al. 2011), while others find poorer planning performance in ASD (e.g. Brunsdon et al. 2015). Systematic, narrative, reviews of planning studies agree that planning performance is impaired in people with ASD. Furthermore, they conclude that the inconsistencies partly reflect the true heterogeneity of the autism spectrum, but might also be due to other factors (Hill 2004; Kenworthy 2008; Sergeant et al. 2002). Three of such factors are emphasized, namely age, task-type and intellectual ability.

Firstly, inconsistencies could be explained by possible age-related changes in planning performance (e.g. Hill 2004). Planning, as well as other executive functions, is related to the frontal striatal brain network (Burgess et al. 2005; Mesulam 2002). This network undergoes intense structural and functional changes from childhood to adolescence, which typically goes hand in hand with age-related improvement in planning ability (Best et al. 2009), with a peak around young adulthood (Anderson et al. 2001; for a meta-analysis see; Romine and Reynolds 2005). This developmental pattern is also experienced in daily life by typically developing individuals and reported by their caregivers (Huizinga et al. 2006; Huizinga and Smidts 2011). Little is known, however, about the development of planning ability in people with ASD. With respect to planning tasks, some studies find age-related improvements from childhood to adolescence (e.g. Happé et al. 2006; Pellicano 2010), whereas other find no gains during this transition (e.g., Goldberg et al. 2005; Van Eylen et al. 2015). However, it has been argued (e.g. Luna 2007; Ozonoff and McEvoy 1994) that people with ASD follow a different developmental trajectory with respect to planning than typically developing people, and, thus, age may explain variability across studies in comparing these groups on planning performance. In sum, the substantial development within the frontal striatal network, together with the possible differences in developmental trajectories of planning ability in people with and without ASD stress the importance of taking the role of age into account when studying planning.

Secondly, the variety of tasks and dependent measures that are reported may partly explain the heterogeneity in findings of planning performance among people with ASD (Kenworthy 2008; Sergeant et al. 2002). For example, it is suggested that people with ASD perform worse on the standard human-administered neuropsychological tasks (e.g. the Tower of London; Lopez et al. 2005) than on their computer-administered variants (e.g. the CANTAB Stockings of Cambridge subtest; see for a review Kenworthy 2008). This conclusion is, however, tentative, as another study did not find a difference in performance between human and computerized administration of the Tower of London task among people with ASD (Williams and Jarrold 2013). This inconsistency in findings combined with the plethora of planning tasks available, raises the question of which of these tasks is most suitable and robust in its findings with regard to people with and without ASD.

Thirdly, variability in intellectual ability (IQ) is posed as a possible moderator of planning performance among people with ASD (Hill 2004; Kenworthy 2008). Some studies show that group differences between ASD and TD on planning measures are more prominent at lower IQ levels (e.g. Hughes et al. 1994). Also, IQ is sometimes found to be more strongly related to performance on cognitive measures in people with ASD than in TD individuals (Brunsdon et al. 2015). However, to date, no systematic review has investigated the role of IQ in planning performance among people with ASD as compared to TD people.

Based on the above, it seems imperative to systematically review the literature on planning ability and articulate the magnitude of the supposed planning deficits in ASD across the lifespan. Furthermore, it seems valuable to investigate other sources of inconsistencies such as the variety of tasks and dependent measures that are reported and the range of intelligence across groups. To this end, this study provides the first comprehensive quantitative review of the literature across all, to the best of our knowledge, studies of planning performance in ASD that fall within our inclusion criteria. By means of a meta-analysis and meta-regression, we aim (1) to present the magnitude of possible planning performance deficits in ASD; (2) to describe potential developmental changes in planning performance across the lifespan; (3) to conceptualize which of the several planning measures is most consistent (e.g. robust) in its findings when comparing people with and without ASD; (4) to investigate whether intelligence levels have an effect on the observed findings when comparing people with and without ASD on planning performance.

## Methods

### Literature Search Strategy

In May and November 2015, a systematic literature search was performed using the online databases PsycINFO, Web of Science, and PubMed. PsycINFO was chosen because it is most frequently used within the behavioral and social sciences and indexes many psychology journals. Web of Science was selected because of its interdisciplinary nature and the high quality of the indexed journals. Finally, given that ASD is seen as a psychiatric disorder and highly comorbid with various medical conditions, PubMed was included to cover the medical journals.^1^ PubMed is one of the biggest and most widely used medical databases that largely indexes psychiatry. The search was done with the following terms of interest related to ASD *(autism; autistic disorder; pervasive developmental disorder; Asperger; ASD; PDD-NOS)* combined with terms related to planning *(planning; executive function; Tower; Tower of London (ToL); Tower of Hanoi (ToH); Stockings of Cambridge (SoC); Behavioral Assessment of the Dysexecutive Syndrome (BADS); Mazes; CANTAB; WISC; NEPSY; D-KEFS; BRIEF)*. Reference lists of selected papers were also checked in search of relevant studies.

### Eligibility Criteria

Studies were only included if they met the following eligibility criteria: (1) ASD participants were the population being studied and they met diagnostic criteria according to the DSM-III, DSM-III-R, DSM-IV, DSM-IV-TR, DSM-5, or ICD-10 (defined by clinical diagnosis, autism questionnaires, interviews or observation schedules: please see Table 1 for details); (2) a typically developing (TD) comparison group was included (3) experimental or clinical neuropsychological planning tasks were used;^2^ (4) studies provided outcome data sufficient and suitable for the calculation of effect sizes, either in the published study or upon request; (5) articles presented original data; (6) studies were written in English and published in a peer-reviewed journal between 2003 and November 2015. Preceding studies on planning performance in ASD were included based on the reviews by Hill (2004) and Sergeant et al. (2002) if they met our eligibility criteria.

### Study Selection

**Fig. 1:**
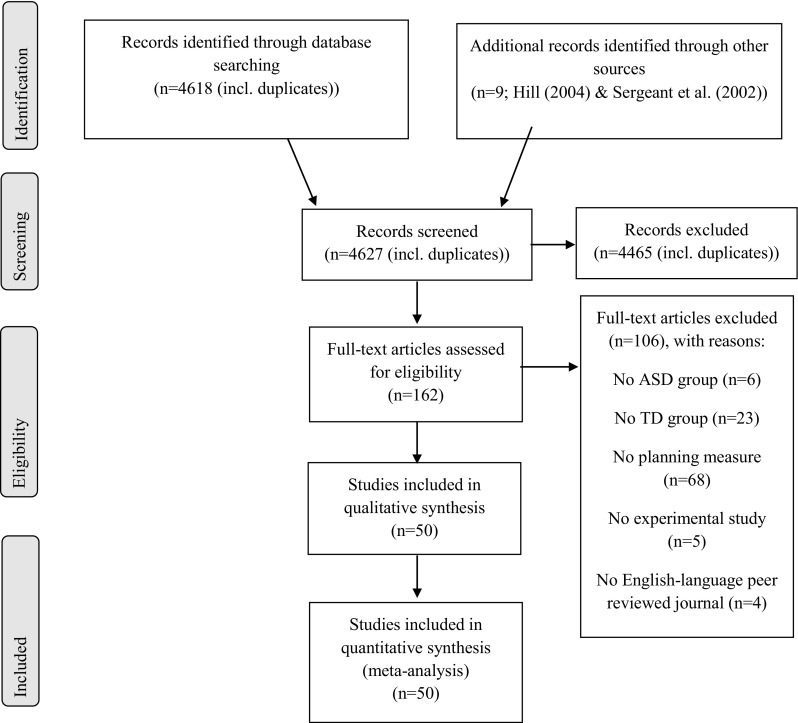
Flow diagram: meta-analysis of planning performance in people with ASD. Six additional studies were excluded from the synthesis because they provided insufficient data to estimate effect sizes after contacting the corresponding authors

Titles and abstracts of retrieved records were screened for eligibility. Studies were excluded if they clearly did not meet our inclusion criteria. After this initial search, the full texts of the remaining records were screened for eligibility. The corresponding authors of articles that did not report sufficient data for the calculation of effect sizes and/or the moderator analysis were contacted to try to retrieve the missing data, as well as any unpublished data on the subject. None of the replies included such unpublished data. Studies that fulfilled the criteria (either immediately or after receiving additional data from the corresponding authors) were included in the meta-analysis. An independent researcher checked the full text screening and the extracted data of the selected studies. Any disagreements between the first author and this researcher were discussed and resolved with a third assessor. See Fig. 1 for a flow diagram of the search results.

**Table 1:** Studies discussing planning in participants with autism spectrum disorders in comparison with typically developing control groups

Study by	Subjects M/F^a^	Age range/ M(SD)	IQ range/ M(SD)	Group assignment ASD	Planning task	Measurement	E
Bölte et al. (2011)	ASD 35/21	14.2 (2.9)	IQ ≥ 70 PIQ: 99.2 (10.6)	Q: - SI: ADI-R, ADOS NSCA: clinical diagnosis CLAS: ICD-10	ToH	Total moves	*g* = −0.19
	TD 23/35	14.6 (4.7)	PIQ: 103.5 (13.1)				
In this study, unaffected siblings of the ASD group formed the comparison group (TD)							
Boucher et al. (2005)	HFA 10/0	23.8 (7.8)	IQ ≥ 70 VIQ: 105.5 (20.2) PIQ: 90.3 (19.3)	Q: modified WADIC SI: - NSCA: clinical diagnosis CLAS: DSM-IV	Zoo Map test *(with MRI)*	Total score	*g* = 0.76
	TD 10/0	24.2 (8.1)	VIQ: 104.4 (13.2) PIQ: 97.5 (16.9)				
Bramham et al. (2009)	ASD 38/7	32.8 (12.5)	IQ ≥ 70 FSIQ: 107 (16.4) VIQ: 106.5 (17.4) PIQ: 105.7 (17.7)	Q: - SI: ADI-R NSCA: clinical diagnosis CLAS: ICD-10	Zoo Map test	Accuracy Map 1	*g* = 0.19
	TD 23/8	32.8 (9.0)	FSIQ: 109.8 (16.8) VIQ: 107.7 (15.8) PIQ: 111 (18.5)		Key Search test	Total score	
Brunsdon et al. (2015)	ASD 150/31	12.1–16.3/13.5 (0.7)	FSIQ: 49–128/ 90 (20.3)	Q: CAST SI: DAWBA (P), ADI-R, ADOS NSCA: clinical diagnosis CLAS: DSM-IV	Planning drawing task, part B (planning)	Planning error score	*g* = 0.43
	TD 110/50	10.9–15.6/12.8 (1.1)	FSIQ: 56–142/ 101.9 (15.1)				
Corbett et al. (2009)	ASD 17/1	7–12/ 9.4 (1.9)	IQ ≥ 70 FSIQ: 94.2 (17.8)	Q: - SI: ADI-R, ADOS NSCA: clinical diagnosis CLAS: DSM-IV-TR	SoC	Total perfect solutions	*g* = 0.91
	TD 12/6	7–12/ 9.6 (1.8)	FSIQ: 112.2 (14.8)				
Geurts et al. (2004)	HFA 41/0	6–13/ 9.4 (1.8)	IQ ≥ 80 FSIQ: 98.3 (18.4)	Q: - SI: ADI-R, DISC-IV (P) NSCA: - CLAS: DSM-IV, ICD-10	ToL	ToL score	*g* = 0.78
	TD 41/0	6–13/ 9.1 (1.7)	FSIQ: 111.5 (18)				
Geurts & Vissers (2012)	ASD 18/5	51–83/ 63.6 (7.5)	DART-IQ: 109.5 (10.3)	Q: SRS SI: - NSCA: clinical diagnosis CLAS: DSM-IV	ToL-Dx	Excess moves	*g* = -0.23
	TD 18/5	51–83/ 63.7 (8.1)	DART-IQ: 109.8 (7.9)				
Goldberg et al. (2005)	HFA 13/4	8–12/ 10.3 (1.8)	IQ ≥ 75 FSIQ: 96.5 (15.9)	Q: - SI: ADI-R, ADOS, ADOS-G NSCA: clinical diagnosis CLAS: DSM-IV	SoC	Total perfect solutions	*g* = 0.56
	TD 21/11	8–12/ 10.4 (1.5)	FSIQ: 112.6 (12.1)				
Griebling et al. (2010)	HFA 35/2	8–45/ 19.1 (9.0)	FSIQ: 104 (15)	Q: - SI: ADI-R, ADOS NSCA: clinical diagnosis CLAS: -	ToH (*with MRI*)	Total moves	*g* = 0.95
	TD 36/2	8–45/ 18.8 (9.0)	FSIQ: 104 (10)				
Hanson & Atance (2014)^b^	ASD 22/3	3.2–8.3/ 5.9 (1.5)	FSIQ: 42–121/ 85.7 (21)	Q: CARS-II SI: - NSCA: clinical diagnosis CLAS: DSM-IV-TR	ToH	Highest level achieved	*g* = 0.09
	TD 22/3	3.1–5.9/ 4.9 (0.9)	FSIQ: 97–128/ 109.1 (8)		Truck loading	Highest level achieved	
Happé et al. (2006)	ASD 32/0	8–16/ 10.9 (2.4)	IQ ≥ 69 FSIQ: 99.7 (18.7) VIQ: 102.4 (18.1) PIQ: 96.6 (17.9)	Q: - SI: - NSCA: clinical diagnosis CLAS: DSM-IV	SoC	Total perfect solutions	*g* = 0.19
	TD 32/0	8–16/ 11.2 (2.0)	FSIQ: 106.8 (13.4) VIQ: 109.8 (12.2) PIQ: 101.7 (18.2)				
Hill & Bird (2006)	AS 16/6	16–61/ 31.1 (13.1)	FSIQ: 80–135/ 110.5 (18.2)	Q: AQ SI: - NSCA: clinical diagnosis CLAS: DSM	Zoo Map test	Accuracy Map 1	*g* = 0.39
	TD 14/8	18–64/ 33.5 (14.5)	FSIQ: 79–135/ 107.9 (14.9)		Key Search test	Total score	
Hughes et al. (1994)	ASD 30	8–19/ 13.2	*Not assessed*	Q: AQ SI: - NSCA: clinical diagnosis CLAS: DSM-III	SoC	Decision time	*g* = −0.43
	TD 44	5–10/ 8.0					
Joseph et al. (2005)	ASD 32/5	5.5–11.1/ 7.9 (1.8)	DAS FSIQ: 57–141/ 87.1 (19.9) DAS VIQ: 61–133/ 87 (19) DAS NVIQ: 49–153/ 91 (22)	Q: - SI: ADI-R, ADOS NSCA: clinical diagnosis CLAS: DSM-IV	Tower (NEPSY)	Total perfect solutions	*g* = 0.51
	TD 24/7	5.1–11.7/ 8.3 (2.1)	DAS FSIQ: 61–117/ 89.8 (14.3) DAS VIQ: 64–122/ 88 (13) DAS NVIQ: 50–114/ 91 (17)				
Kaufmann et al. (2013)	AS 8/2	14.7 (5.0)	FSIQ: 102.3 (15.9) VIQ: 107.6 (13.2) PIQ: 95.8 (16.6)	Q: - SI: ADI-R, ADOS NSCA: clinical diagnosis CLAS: DSM-IV-TR	SoC *(with MRI)*	Total perfect solutions	*g* = −0.04
	TD 8/2	13.8 (5.3)	FSIQ: 109.5 (6.4) VIQ: 114 (9.9) PIQ: 106 (10.6)				
Keary et al. (2009)	ASD 29/3	8.8–45.7/ 9.8 (10.2)	IQ ≥ 70 75–135/ FSIQ: 102.9 (13.6) VIQ: 106.9 (15.6) PIQ: 97.8 (12.5)	Q: - SI: ADI-R, ADOS NSCA: clinical diagnosis CLAS: DSM-IV	ToH *(with MRI)*	Total moves	*g* = 0.93
	TD 31/3	9.2–43.9/ 18.6 (9.1)	86–121/ FSIQ: 104 (10.5) VIQ: 104.7 (10.4) PIQ: 102.6 (10)				
Kimhi et al. (2014)	ASD 25/4	3–6/ 4.9 (0.9)	FSIQ: 103.5 (17.2)	Q: - SI: ADI-R NSCA: clinical diagnosis CLAS: DSM-IV-TR	ToL	Total perfect solutions	*g* = 0.58
	TD 26/4	3–6/ 4.6 (0.9)	FSIQ: 107.6 (14.1)				
Landa & Goldberg (2005)	HFA 19	7.3–17.3/ 11.0 (2.9)	IQ ≥ 80 81–139/ FSIQ: 109.7 (15.8) VIQ: 113.5 (17.1) PIQ: 104.6 (13.5)	Q: - SI: ADI-R, ADOS (-G) NSCA: - CLAS: -	SoC	Total perfect solutions	*g* = 1.01
	TD 19	7.2–17.2/ 11.0 (2.9)	90–138/ FSIQ: 113.4 (14.3) VIQ: 115.6 (15.8) PIQ: 108.5 (12.1)				
Limoges et al. (2013)	ASD 16/1	16–27/ 21.7 (3.5)	FSIQ: 89–129/ 104.1 (11.3) VIQ: 103.2 (16.2) PIQ: 103.5 (13.1)	Q: - SI: ADI-R, ADOS NSCA: clinical diagnosis CLAS: DSM-IV	ToL *(with EEG)*	Total perfect solutions (%)	*g* = 0.64
	TD 13/1	16–27/ 21.8 (4.1)	FSIQ: 92–124 112.3 (9.8) VIQ: 113 (9.6) PIQ: 112.1 (10.9)				
Lopez et al. (2005)	ASD 14/3	19–42/ 29.0	PIQ ≥ 70 FSIQ: 77 (15) VIQ: 73 (16) PIQ: 84.1 (12.2)	Q: GARS (P) SI: ADI- R, ADOS NSCA: clinical diagnosis CLAS: DSM-IV	Tower of California (D-KEFS)	Total constructed towers	*g* = 1.15
	TD 11/6	18–45/ 29.0	FSIQ: 89 (13) VIQ: 92 (15) PIQ: 87.6 (11.7)				
Losh et al. (2009)	HFA 29/7	21.5 (5.5)	IQ ≥ 80 FSIQ: 101.2 (18.1)	Q: - SI: ADI- R, ADOS NSCA: clinical diagnosis CLAS: DSM-IV	ToH	Total moves	*g* = 0.27
	TD 34/7	23.4 (5.6)	FSIQ: 108.3 (15)				
Low et al. (2009)	ASD 23/4	5.3–13.1/ 8.3 (2.2)	*Not assessed*	Q: - SI: - NSCA: clinical diagnosis CLAS: DSM-IV	Mazes	Accuracy	*g* = 0.63
	TD 23/4	4.5–10.7/ 6.6 (1.3)					
McCrimmon et al. (2012)	AS 26/7	16–21/ 18.8 (1.6)	IQ ≥ 85 FSIQ: 113.2 (10.6) VIQ: 114.1 (12.2) PIQ: 108.9 (9.9)	Q: - SI: - NSCA: clinical diagnosis CLAS: DSM-IV-TR	Tower (D-KEFS)	Total score	*g* = 0.07
	TD 26/7	16–21/ 18.9 (1.6)	FSIQ: 110.1 (8.8) VIQ: 109 (10.7) PIQ: 108.7 (10)				
Medeiros & Winsler (2014)	ASD 26/1	7–18/ 11.9 (2.7)	*Not assessed*	Q: - SI: - NSCA: clinical diagnosis CLAS: DSM-IV	ToH-Revised	Total moves	*g* = 0.51
	TD 18/8	7–18/ 10.3 (3.2)					
Ozonoff & Jensen (1999)	ASD 40	12.6 (3.4)	IQ ≥ 70 FSIQ: 95.2 (18.8) VIQ: 93.3 (20.0) PIQ: 98.6 (19.8)	Q: - SI: ADI-R, ADOS NSCA: clinical diagnosis CLAS: DSM-IV	ToH	Total score	*g* = 0.70
	TD 29	12.1 (3.0)	FSIQ: 107.8 (10.8) VIQ: 107.8 (12.3) PIQ: 106.8 (12.5)				
Ozonoff et al. (2004)	ASD 72/7	6–47/ 15.7 (8.7)	FSIQ: 106.3 (16.3) VIQ: 104.9 (17.9) PIQ: 106 (16)	Q: - SI: ADI-R, ADOS-G NSCA: clinical diagnosis CLAS: ICD-10	SoC	Total perfect solutions	*g* = 0.87
	TD 58/12	6–47/ 16.0 (7.6)	FSIQ: 106 (11.5) VIQ: 106.1 (11.6) PIQ: 105 (12)				
Panerai et al. (2014)	HFA 9/2	8.9 (3.1)	IQ ≥ 85 85–111	Q: - SI: - NSCA: clinical diagnosis CLAS: DSM-IV-TR	ToL	Total perfect solutions	*g* = 1.79
	TD 6/3	9.7(2.6)	*Not assessed within study*				
Pellicano et al. (2006)	ASD 35/5	4.1–7.3/ 5.6 (0.9)	IQ ≥ 80 VIQ (PPVT): 82–122/ 101.2 (11) PIQ (Leiter): 83–141/ 113.6 (14.1)	Q: SCQ (P) SI: ADI-R NSCA: clinical diagnosis CLAS: DSM-IV/ICD-10	Mazes	Accuracy	*g* = 0.63
	TD 31/9	4-7.3/ 5.5. (0.9)	VIQ (PPVT): 75–121/ 103.3 (9.9) PIQ (Leiter): 91–143/ 112.52 (14.47)		ToL	Total perfect solutions	
Verbal (VIQ) and nonverbal IQ (PIQ) were assessed with the Peabody Picture Vocabulary Test (PPVT) and the Leiter International Performance Scale (Leiter), which does not allow an estimation of total IQ (FSIQ)							
Pellicano (2007)	ASD 25/5	4.1–7.3/ 5.6 (0.9)	VIQ (PPVT): 85–122/ 100 (10.6) PIQ (Leiter): 85–141/ 113.9 (13.7)	Q: SCQ (P) S: ADI-R NSCA: clinical diagnosis CLAS: DSM-IV	Mazes	Accuracy	*g* = 0.54
	TD 31/9	4-7.3/ 5.5 (0.9)	VIQ (PVVT): 75–121/ 103.3 (9.9) PIQ (Leiter): 91–143/ 112.5 (14.5)		ToL	Total perfect solutions	
VIQ and PIQ were assessed with the PPVT and the Leiter, which does not allow an estimation of FSIQ							
Pellicano (2010)	ASD 40/5	T1: 4.1–7.3/ 5.6 (0.9)	IQ ≥ 80 T1: VIQ: 80–122/ 97.1 (11.5) PIQ: 83–141/ 113.3 (13.9)	Q: - SI: ADI-R, ADOS-G NSCA: clinical diagnosis CLAS: DSM-IV	ToL	T1: total perfect solutions	*g* = 1.54
	TD 37/8	T1: 4-7.3/ 5.4 (0.9)	T1: VIQ: 87–120/ 100.9 (8.7) PIQ: 89–147/ 115.6 (16.4)				
VIQ and PIQ were assessed with the PPVT and the Leiter, which does not allow an estimation of FSIQ							
Planche & Lemonnier (2012)	HFA 14/1 + AS 13/2	6.1–10.2/ 8.4 (1.5)	IQ ≥ 70 FSIQ: 101.8 (21.5)	Q: - SI: ADI-R NSCA: clinical diagnosis CLAS: ICD-10	Tower (NEPSY)	Total score	*g* = −0.04
	TD 12/3	6–10/ 9.1 (1.4)	FSIQ: 106.2 (8.3)				
Prior & Hoffmann (1990)	ASD 9/3	10.2–17.3/ 3.8	FSIQ (Leiter): 76–109/ 88	Q: - SI: - NSCA: clinical diagnosis CLAS: Rutter (1978)	Milner mazes	Number of errors	*g* = 1.32
	TD 9/3	10.3–17/ 13.8	FSIQ (Leiter): 85–112 / 100				
Rajendran et al. (2005)	ASD 8/4	11.4/ 16.5 (6.8)	FSIQ: 102 (21.5) VIQ: 110.3 (22.5) PIQ: 93.3 (22.8)	Q: - SI: - NSCA: clinical diagnosis CLAS: DSM-IV-TR	Zoo Map test	Summary profile score	*g* = 0.68
	TD 8/4	12–39/ 16.8 (7.4)	FSIQ: 109 (13) VIQ: 111.8 (14.3) PIQ: 104.5 (14.4)		Key Search test	Summary profile score	
Rajendran et al. (2011)	ASD 16/2	11.6–17.4/ 13.9 (1.7)	FSIQ: 96.2 (13.1) VIQ: 106.2 (14.6) PIQ: 87.6 (14.8)	Q: SCQ (P) SI: - NSCA: clinical diagnosis CLAS: DSM-IV-TR	Six Elements test	Summary profile score	*g* = 0.85
	TD 14/4	12.2–18.3/ 13.8 (1.4)	FSIQ: 106.8 (10) VIQ: 106.4 (12.2) PIQ: 106.1 (8.9)				
Robinson et al. (2009)	ASD 42/12	8–17/ 12.5 (2.8)	IQ ≥ 70 FSIQ: 103.5 (10.5)	Q: SCQ (P) SI: - NSCA: clinical diagnosis CLAS: DSM-IV	ToL	Total moves	*g* = −0.53
	TD 42/12	8–17/ 12.1 (2.3)	FSIQ: 104.8 (9.1)				
Sachse et al. (2013)	HFA 27/3	14–33/ 19.2 (5.1)	IQ ≥ 70 FSIQ: 105.3 (12.3)	Q: - SI: ADI-R. ADOS NSCA: - CLAS: DSM-IV-TR	SoC	Total perfect solutions	*g* = 0.37
	TD 24/4	14–33/ 19.9 (3.6)	FSIQ: 109.3 (11.5)				
Schurink et al. (2012)	PDD-NOS 19/9	7–12/ 10.5 (1.4)	IQ ≥ 70 FSIQ: 81.4 (8.4)	Q: CSBQ (P) SI: - NSCA: clinical diagnosis CLAS: DSM-IV-TR	ToL	ToL score	*g* = 0.60
	TD 19/9	7–12/ 10.4 (1.3)	*Not reported*				
Semrud-Clikeman et al. (2010)	ASD 8/7	9.1–16.5/ 10.6 (2.6)	IQ ≥ 80 FSIQ: 100.8 (13)	Q: - SI: ADI-R, ADOS NSCA: clinical diagnosis CLAS: DSM-IV	Tower (D-KEFS)	Total achievement	*g* = 0.82
	TD 23/9	9.1–16.5/ 9.8 (2.1)	FSIQ: 109.4 (10)				
Sinzig et al. (2008)	ASD 16/4	8.3–18.9/ 14.3 (3.0)	IQ ≥ 80 PIQ: 112 (17.7)	Q: - SI: ADI-R, ADOS NSCA: clinical diagnosis CLAS: DSM-IV-TR	SoC	Total perfect solutions	*g* = 0.07
	TD 14/6	7.6–17.6/ 13.1 (3.0)	PIQ: 113 (11.9)				
IQ (nonverbal) was measured using the Culture Fair Intelligence Test, which only assesses nonverbal IQ (PIQ)							
Taddei & Contena (2013)	ASD 30/8	13.1 (3.3)	*Not assessed*	Q: - SI: - NSCA: clinical diagnosis CLAS: DSM-IV-TR, ICD-10	Cognitive Assessment System (CAS) - Planning	Total score	*g* = 2.27
	TD 10/5	12 (2.85)					
Unterrainer et al. (2015)	ASD 18	10.1 (2.4)	IQ ≥ 70 FSIQ: 97.1 (16.4)	Q: SRS SI: ADOS-G, ADI-R NSCA: clinical diagnosis CLAS: DSM-IV-TR/ ICD-10	ToL (computerized)	Total perfect solutions	*g* = 0.13
	TD 42	9.8 (2.4)	FSIQ: 97.6 (13.9)				
Van Eylen et al. (2015)	ASD 30/20	8–18/ 12.2 (2.6)	IQ ≥ 70 FSIQ: 104.3 (10.8) VIQ: 104.3 (15.9) PIQ: 104.3 (13.2)	Q: SRS SI: 3Di NSCA: clinical diagnosis CLAS: DSM-IV-TR	Tower (D-KEFS)	Total score	*g* = 0.20
	TD 30/20	8–18/ 12.5 (2.7)	FSIQ: 107.7 (9.3) VIQ: 111.6 (11.4) PIQ: 103.8 (13.7)				
Verté et al. (2005)	HFA 57/4	6–13/ 9.1 (1.9)	IQ ≥ 80 FSIQ: 99.2 (17.1)	Q: - SI: ADI-R, DISC-IV NSCA: clinical diagnosis CLAS: DSM-IV	ToL	ToL score	*g* = 0.82
	TD 40/7	6–13/ 9.4 (1.6)	FSIQ: 112.1 (9.7)				
Verté et al. (2006)	ASD 99/13	6–13/ 8.6 (1.8)	IQ ≥ 80 FSIQ: 100.6 (16) VIQ: 97.3 (17.6) PIQ: 104.6 (17.6)	Q: - SI: ADI-R, DISC-IV NSCA: clinical diagnosis CLAS: DSM-IV-TR	ToL	ToL score	*g* = 0.68
	TD 40/7	6–13/ 9.4 (1.6)	FSIQ: 112.1 (9.7) VIQ: 113.6 (10.4) PIQ: 108.5 (11.9)				
Wallace et al. (2009)	ASD 26/2	12–20/ 15.7 (2.1)	IQ ≥ 80 FSIQ: 110.3 (16.8) VIQ: 109.7 (17.1) PIQ: 108.8 (16.8)	Q: - SI: ADI-R, ADOS NSCA: clinical diagnosis CLAS: DSM-IV	ToL-Dx	Excess moves	*g* = 0.63
	TD 24/1	12–19/ 16.4 (1.8)	FSIQ: 113.8 (10) VIQ: 111.9 (10.8) PIQ: 112.5 (10.3)				
White et al. (2009)	ASD 41/4	7–12/ 9.6 (1.4)	FSIQ: 105.9 (12.1) VIQ: 111 (14.7) PIQ: 98 (11.2)	Q: - SI: 3Di NSCA: clinical diagnosis CLAS: -	Zoo Map test	Accuracy Map 1	*g* = 0.41
	TD 21/6	7–12/ 9.9 (1.3)	FSIQ: 110.7 (14.6) VIQ: 115 (15.8) PIQ: 103 (12.4)		Key Search test	Total score	
Williams & Jarrold (2013)	ASD 21	10.45 (2.10)	VIQ: 103.3 (18) PIQ: 110 (16.4)	Q: SRS (P) SI: 3Di NSCA: clinical diagnosis CLAS: DSM-IV-TR, ICD-10	ToL	Total moves *(manual version)*	*g* = 0.59
	TD 22	10.61 (1.3)	VIQ: 105.6 (13.3) PIQ: 107.2 (13)				
Participants also completed a computerized version of the ToL, which gave the same results (ns)							
Williams et al. (2012)	ASD 17	42.13	FSIQ: 114 (13.4) VIQ: 112.8 (11.8) PIQ: 112.8 (15.3)	Q: AQ SI: ADOS NSCA: clinical diagnosis CLAS: DSM-IV-TR, ICD-10	ToL	Total moves silent condition	*g* = 0.26
	TD 17	39.43	FSIQ: 116.7 (13.3) VIQ: 117.6 (13.1) PIQ: 112.6 (11.1)				
Please note that for the ToL test, n_ASD_ = 15 and n_tD_ = 16							
Williams et al. (2014)^b^	ASD 65	8–46/ 18.8 (9.7)	FSIQ: 98.8 (14) VIQ: 102 (15.6)	Q: - SI: ADI-R, ADOS NSCA: clinical diagnosis CLAS: DSM-IV-TR	ToH	Total moves	*g* = 0.24
					Zoo Map test	Summary profile score	
	TD 65	8–46/ 19.2 (10.1)	FSIQ: 102.1 (8.8) VIQ: 102.6 (8.9)		Key Search test	Summary profile score	
Zinke et al. (2010)	HFA 13/2	7–12/ 9.0 (1.5)	≥ 78 96.4 (14.5)	Q: - SI: ADI-R, ADOS NSCA: - CLAS: ICD-10	ToL	Total perfect solutions	*g* = 0.98
	TD 14/3	6–12/ 9.8 (1.7)	*Not reported*				

*A* Author; *ADI-R* Autism Diagnostic Interview Revised; *ADOS(-G)* Autism Diagnostic Observation Schedule(-Generic); *AS* Asperger Syndrome; *ASD* Autism spectrum disorder (could include autism, Asperger syndrome or PDD-NOS); *AQ* Autism Spectrum Questionnaire; *CARS-II* Childhood Autism Rating Scale, second edition; *CAST* Childhood Autism Spectrum Test; *CLAS* Classification system used; *CSBQ (P)* Children’s Social Behavior Questionnaire (Parent version); *DART* Dutch Adult Reading Test; *DAS* Differential Ability Scales; *DAWBA* Development and Wellbeing Assessment; *DISC-IV (P*) Diagnostic Interview Schedule for Children for DSM-IV, (parent version); *D-KEFS* Delis-Kaplan Executive Function System; *DSM-IV(-TR)* Diagnostic and Statistical Manual of Mental Disorders, fourth edition, (text-revised); *F* Female; *FSIQ* Full Scale Intelligence Quotient; *GARS* Gilliam Autism Rating Scale; *HFA* High Functioning Autism; *ICD-10*; International statistical classification of diseases and related health problems, tenth edition; *IQ* Intelligence Quotient; *M* male; *NEPSY* Developmental NEuroPSYchological Assessment; *ns* Did not reach statistical significance; *NSCA* Nonstructural clinical assessment; *P* Parent; *PIQ* Performance Intelligence Quotient; *Q* Questionnaire; *RT* Reaction Time; *SCQ* Social Communication Questionnaire; *SI* Structured instrument such as specially developed standardized interviews and observation schedules; *SoC* Stockings of Cambridge; *SRS* Social Responsiveness Scale; *TD* Typically developing group; *ToH* Tower of Hanoi; *ToH-Revised* Tower of Hanoi-Revised; *ToL* Tower of London; *ToL-Dx* Tower of London-Drexel; *VIQ* Verbal Intelligence Quotient; *WADIC* Wing’s Autistic Disorder Interview Checklist; *3Di* Developmental, Dimensional and Diagnostic Interview

^a^If only one digit is reported, this refers to the total sample size because the division of gender (number of males and females) was unknown

^b^When multiple planning tasks of different type of tasks were assessed within the same study, we chose type of task (Tower, BADS, CANTAB) for the moderator analysis of task-type based on the highest number of similar type of task available (e.g., Williams et al. (2014) is categorized as BADS)

### Data Collection Process

This study followed the Preferred Reporting Items for Systematic Reviews and Meta-Analysis Protocol (PRISMA-P) flow diagram and checklist (Moher et al. 2015). The literature search generated 4618 hits; an additional nine articles was screened for eligibility from the reviews by Hill (2004) and Sergeant et al. (2002). Based on titles and abstracts, the number of articles was narrowed down to 162 studies. After full text screening, 106 studies did not meet inclusion criteria according to the first author and an independent researcher. Reasons for excluding studies were the absence of an ASD group (*n* = 6) or TD comparison group (*n* = 23), no assessment of an experimental or clinical neuropsychological planning task (*n* = 68), the non-experimental nature of the study (e.g. a review or case report; *n* = 5) or the study was not published in an English-language peer reviewed journal (*n* = 4).

Of the 56 studies that met inclusion criteria, 7 studies reported insufficient information to calculate the effect size (Booth et al. 2003; Just et al. 2007; Lin et al. 2013; McLean et al. 2014; Olivar-Parra et al. 2011; Ruta et al. 2010; Sinzig et al. 2008). Corresponding authors were contacted and one provided the requested information (Sinzig et al. 2008). Therefore, 50 studies were included in our meta-analysis. This resulted in a combined sample size of 1755 participants with ASD and 1642 TD comparison individuals (see Table 1). Twenty-six studies were conducted with childhood samples (mean age ≤12 years), 11 studies with adolescent (mean age 13–18 years), and 13 studies with adult samples (mean age: >18 years). All the study information listed in Table 1 was first recorded by the first author and then verified by an independent researcher.

### Dependent Variables

We recorded the dependent measure for each task. It is important to note that despite the use of similar tasks, the studies differed considerably in the reported dependent measure. In addition, the majority of studies reported more than one dependent measure for the task of interest. Therefore, we selected the measure that best reflected planning, and was most commonly reported among the included studies. If this measure was not reported, we requested this data from the corresponding author or, if not available upon request, selected the next measure most demonstrative of planning. When two or more dependent variables were considered to reflect this equally, we tried to reduce heterogeneity by selecting the variable most frequently reported in other included articles. The selection of dependent measures was made before effect sizes were calculated to minimize experimenter bias. Eight studies presented multiple planning tasks. To prevent dependency in our data and extra weight being assigned to these studies in the meta-analysis, we chose to combine these effect sizes within the same study into one effect size per study (Borenstein et al. 2009), using an earlier reported inter-test correlation (range 0.41–0.63). If this correlation was not available, we used an inter-test correlation of 0.7 as the tasks are supposed to all measure planning ability (rule of thumb in meta-analysis, see Borenstein et al. 2009). See Table 1 for the dependent measure that was selected per task.^3^

For each continuous outcome, a standardized mean difference (Hedges’ *g*; Hedges and Olkin 1985) was calculated—the difference between the mean score of the ASD group and TD group divided by the pooled standard deviation per planning measure in each study (see Table 1). This effect size is widely used, easily interpretable and can be calculated from *t*-test statistics (Borenstein et al. 2009; Turner and Bernard 2006). Effect sizes were interpreted accordingly: *g* = 0.2–0.5 is small; *g* = 0.5–0.8 is medium; *g* > 0.8 is large. Therefore, a smaller Hedges’ *g* stands for a smaller distinction between the ASD and TD group. A positive effect size indicates poorer performance by the ASD group as compared to the TD group, whereas a negative effect size indicates that the ASD group outperformed the TD group.

### Data Analysis

The data were analyzed using the Metafor package for R (Viechtbauer 2010). Variability among the true effect was expected due to differences in methods and sample characteristics between studies. In order to account for this within- and between-study variation, a random effects model was chosen. In this procedure, the effect size is corrected for sample size of each individual study before the weighted average effect size of planning performance across studies is calculated. A significant degree of between-study variation would imply heterogeneity between studies, driven by additional factors other than planning ability. Therefore, the test of homogeneity of effects was performed (*Q* statistic). Since this test does not quantify the amount of between-study variation, we also estimated the amount of residual heterogeneity (τ^2^) and ratio of true to total variance (*I* ^2^). The *I* ^2^ is interpreted as the proportion of the observed variability in a set of effect sizes that reflects real differences among true effects (Borenstein et al. 2009).

Next, random restricted maximum likelihood meta-regression techniques were applied to determine possible moderating effects of age. Age was indexed as the mean age of the ASD participants. Using this same technique IQ was explored. For task-type, a subgroup analysis was performed to compare the mean effect for different subgroups of studies using the same type of planning task [Tower; BADS (BADS Zoo Map test, BADS Key Search test, BADS Six Elements test and Mazes); CANTAB (SoC)]. The effect of each moderator was tested separately. The presence of publication bias was assessed with a funnel plot, a regression test for funnel plot asymmetry, and the Trimm and Fill method (Duval and Tweedie 2000). The fail-safe *N* analysis (Rosenthal 1979) was performed to indicate the robustness of the overall effect.

## Results

### Overall Results of Planning Performance in ASD versus TD

**Fig. 2:**
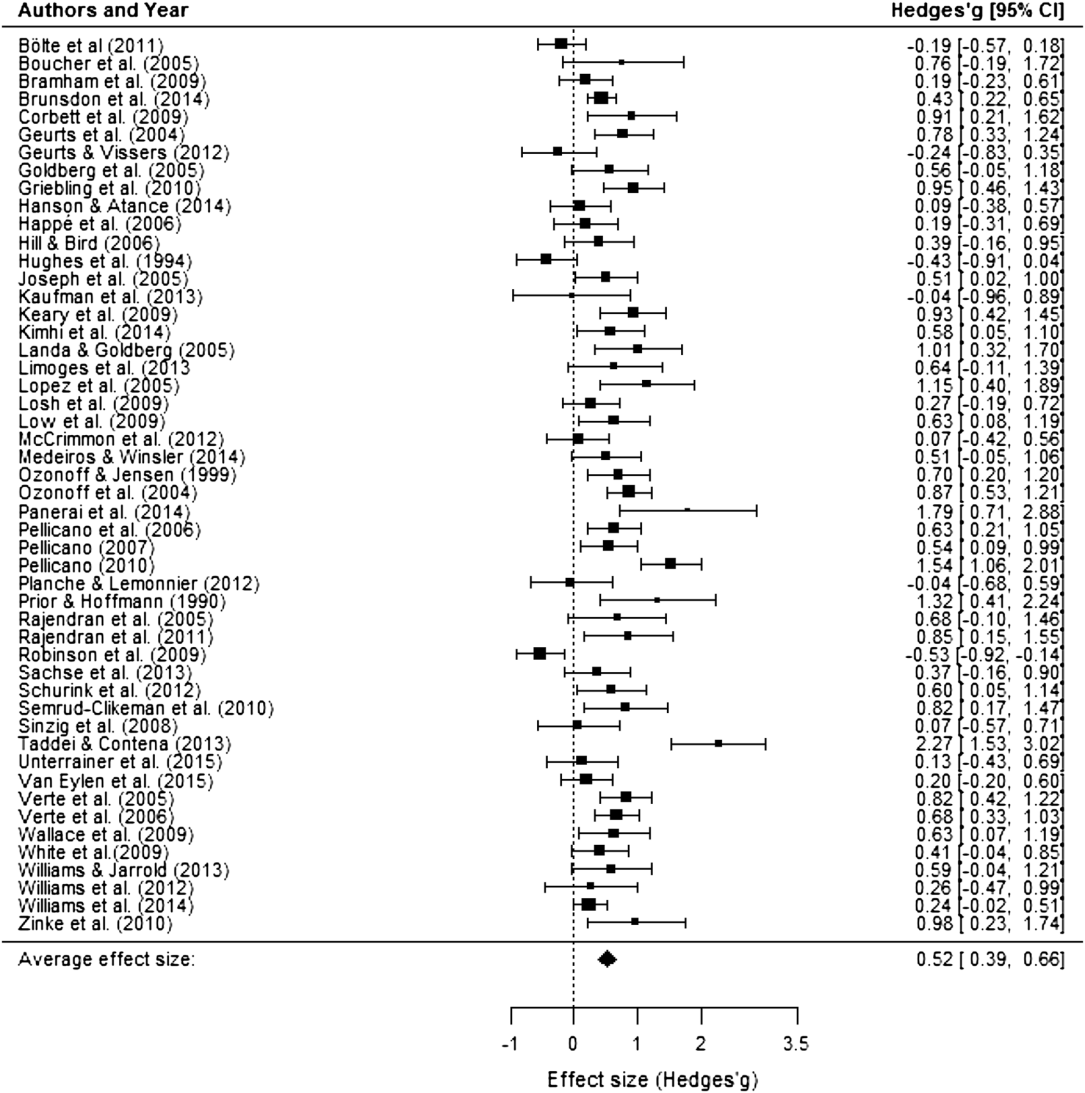
Forest plot indicating effect sizes (Hedges’ *g*) and 95% confidence intervals for each study effect included in the meta-analysis. Positive effect sizes indicate worse planning performance in the ASD group as compared to the TD group while negative effect sizes indicate that the ASD group outperformed the TD group

The random effects meta-analysis showed a significant medium positive effect size (Hedges’*g*) of 0.52 (95% CI 0.39–0.66; range −0.53–2.27), indicating that individuals with ASD perform worse on planning tasks as compared to TD controls (*z* = 7.57, *p* < .0001). As expected, there was significant heterogeneity in effect sizes across planning studies (*Q* (49) = 161.7, *p* < .0001; τ^2^=0.16; *I* ^2^ = 71.43). The forest plot in Fig. 2 depicts the summary effect and individual effect sizes of planning performance by ASD as compared to TD.

### Outliers and Publication Bias

To investigate the presence of influential data points or outliers, we visually inspected the forest plot (Fig. 2) and calculated Cook’s distance. Cook’s distance was below one for all effect sizes which suggests that there were no outliers. In addition, a leave-one-out analysis showed that leaving any study out of the meta-analysis would not change the overall results. Finally, a QQ-plot confirmed that there is a normal distribution of effect sizes. None of these methods thus revealed any outliers.

**Fig. 3:**
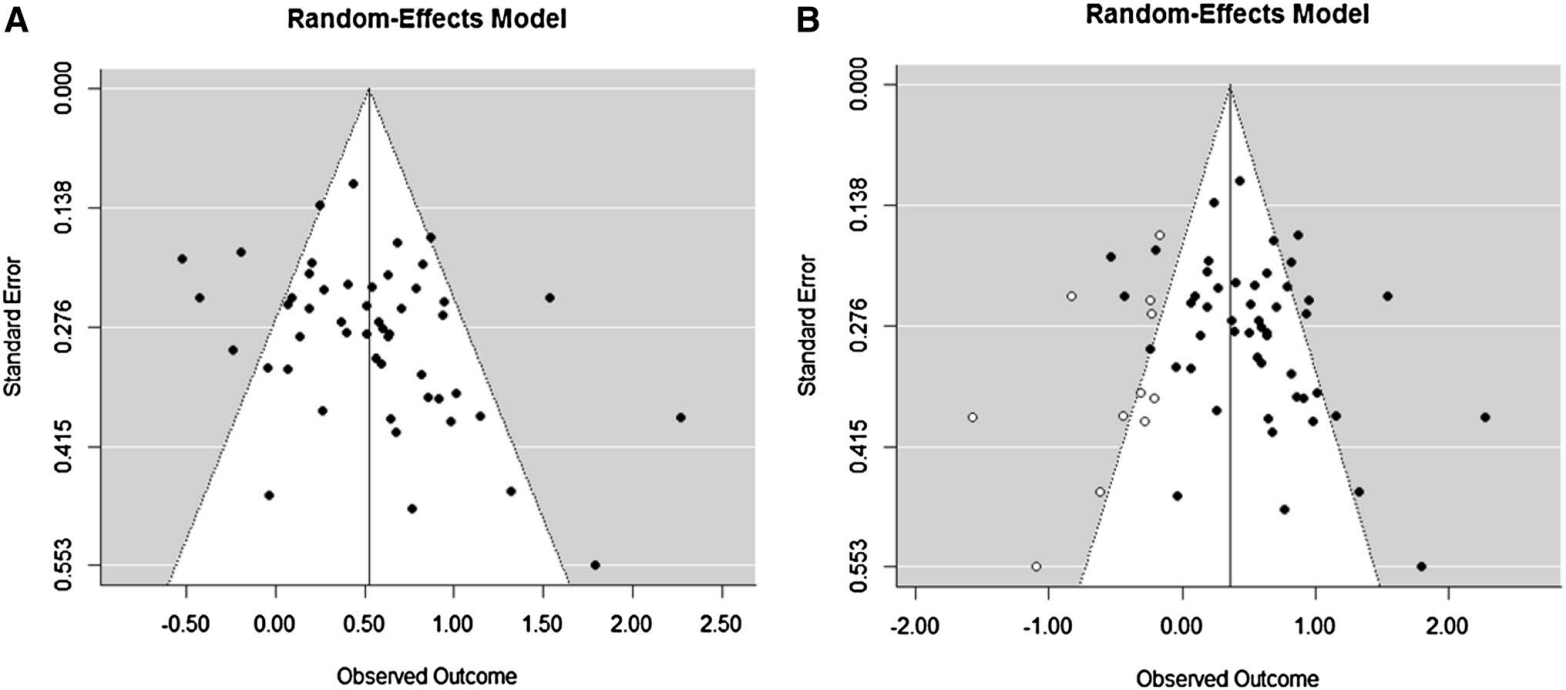
Funnel plots (*panel* **a** original; *panel* **b** including hypothetical missing studies) used to explore publication bias

A regression test for funnel plot asymmetry was significant (*z* = 2.66, *p* = .008), and therefore suggested the presence of publication bias. This was confirmed by the Trimm and Fill method (Duval and Tweedie 2000), which demonstrated that 11 unpublished studies were missing on the left side of the funnel plot (see Fig. 3). Inclusion of these missing studies would decrease the overall summary medium effect size of 0.52 to a small effect size of 0.37 (95% CI 0.21–0.51). The Rosenthal’s fail safe *N* analysis demonstrated the robustness of the overall effect (3401 null findings are needed to nullify the overall significant effect). Hence, the observed overall effect size is still of relevance, but we must consider a moderate impact of publication bias in the research of planning performance in people with ASD as compared to TD individuals.

### Age

Age did not significantly moderate the effect sizes across planning studies (*Q*_AGE_ = 2.89, *p* = .09), and heterogeneity in effect sizes remained significant (Q (48) = 152.65, *p* < .0001; τ^2^ = 0.15; *I* ^2^ = 70.30). Based on the discussion in non-experimental research whether an increase in planning difficulties in ASD can be found around adolescence (Van den Bergh et al. 2014 versus Rosenthal 2013), we also inspected a quadratic relationship. We inserted age as a centered quadratic predictor, and found no support for a quadratic association between age and planning performance (Q_AGE_^2^ = 2.62, *p* = .11). Furthermore, heterogeneity between studies remained significant (*Q* (48) = 156.11, *p* < .0001; τ^2^ = 0.15; *I* ^2^ = 70.74). However, in one study the mean age of the ASD participants deviated far from the grand mean age of ASD participants (Geurts and Vissers 2012). Visual inspection of the corresponding boxplot showed that this study was indeed an outlier. Excluding this study did, however, not alter our age-related findings as age was still not a relevant moderator (linear: Q_AGE_ = 0.72, *p* = .40; quadratic: Q_AGE_^2^ = 0.20, *p* = .65) and heterogeneity in effect sizes remained significant (Q (47) = 152.42, *p* < .0001; τ^2^ = 0.15; *I* ^2^ = 70.89).

### Task-type

The studies were classified according to the following type of tasks: BADS (*n* = 13), CANTAB (*n* = 7) or Tower (*n* = 28). Two studies did not fall in any category and were, therefore, excluded from the moderator analysis (Brunsdon et al. 2015; Taddei and Contena 2013). Task-type was not a significant moderator of effect sizes across planning studies (*Q*_TASK_ = 0.10, *p* = .95) and heterogeneity between studies remained significant (*Q* (45) = 138.73, *p* < .0001; τ^2^ = 0.14; *I* ^2^ = 66.38).

### IQ

Forty out of 50 studies included estimates of total IQ. For IQ, no outliers were detected. IQ did not significantly moderate the effect sizes across these studies (*Q*_IQ_ = 2.56, *p* = .11) and heterogeneity in effect sizes remained significant (*Q* (38) = 94.41, *p* < .0001; τ^2^ = 0.09; *I* ^2^ = 59.92).

## Discussion

The aim of the present meta-analysis was to systematically and quantitatively review the experimental literature on planning performance in ASD to examine whether people with ASD encounter difficulties with this skill. In line with non-experimental research, the meta-analysis revealed that people with ASD indeed show poorer planning performance as compared to typically developing (TD) individuals. This difference was moderate in size and consistent across the lifespan, various types of planning tasks, and different intelligence levels. However, please note that examination of publication bias indicated that there may be missing studies with negative effect sizes (i.e., individuals with ASD outperforming people without ASD with respect to planning) in our meta-analysis. Hence, the true effect size might be smaller, but planning deficits do still seem to exist in people with ASD.

As suggested in one of the last, narrative, reviews on planning performance (Hill 2004), we investigated whether age influenced performance on planning tasks. Despite a rather broad age range across studies (5–64 years of age) and the inclusion of 50 studies, age did not moderate the variability in findings across studies that compared people with ASD to TD individuals on planning ability. More specifically, people with ASD seem to have persistent planning deficits throughout their life, unable to attain the performance level of TD individuals. This in line with previous reports on planning (e.g. O’Hearn et al. 2008), and suggests that the developmental trajectory of people with ASD runs parallel below to the trajectory of TD individuals. To date, only one prospective longitudinal study has examined this trajectory in young children with ASD (4–7.3 years) and found age-related gains in executive functioning (including planning) (Pellicano 2010). Moreover, studies focusing on middle aged and older people with ASD were rather scarce in the current meta-analysis. Hence, longitudinal studies across the whole lifespan are needed to test whether a parallel pattern can be replicated, and to improve our understanding about the developmental trajectory of planning skills in ASD.

In addition, we found that several planning measures seem to be evenly consistent in their findings when comparing people with and without ASD on planning performance. Even though the measures differ from each other in for example difficulty level, instruction, and structure of the task, they all find medium effect sizes—all find a moderate deficit with regard to planning ability in people with ASD as compared to people without ASD. This suggests that, contrary to what some previous reports claimed (e.g. Kenworthy 2008; Sergeant et al. 2002), the task-type cannot explain discrepant findings in the literature. Hence, when focusing on the most commonly used tasks the type of planning task does not seem to be crucial when assessing planning abilities in ASD.

Finally, the variability in effect sizes across studies could not be explained by intelligence level. While IQ is strongly related to general executive functioning (e.g. Dang et al. 2014; Friedman et al. 2006), and therefore planning ability, it does not impact the difference in planning performance between those with and without ASD. Our findings should, however, be interpreted with caution, as the number of included studies in the moderator analysis was smaller than in the overall meta-analysis due to missing IQ estimates in ten studies. Moreover, the majority of the included studies and five of the studies that missed exact IQ estimates only assessed people within the normal intelligence range (IQ > 70). Therefore, our finding may not generalize to lower ranges of IQ. Previous reports show that the effect of intellectual ability on ASD outcome is more pronounced in groups of individuals with ASD with IQs within the lower ranges (Matson and Horovitz 2010; McGovern and Sigman 2005). As IQ is strongly related to executive functioning, findings on planning tasks of these individuals are hard to interpret due to their restricted cognitive abilities. Poor performance on measures of planning in individuals with a low IQ may be, at least in large part, attributable to their below average IQ rather than a planning deficit per se. This is in line with the statement by Hill and Bird (2006) that executive function difficulties that are related causally to ASD are most likely to be found in their most pure forms in individuals with ASD with a higher IQ. Although it should be stressed that it is a limitation that we could not investigate these lower ranges of intellectual ability, the fact that our meta-analysis mostly included individuals with ASD within the normal IQ range strengthens the finding that there is indeed a planning deficit in people with ASD as compared to TD individuals.

Although a difference was observed between people with ASD and TD individuals with respect to planning ability, there was a large amount of heterogeneity in these differences across studies that we could not explain by our pre-specified factors. This complicates the interpretation of our findings as it suggests that there must be additional factors that influence the difference in planning performance among people with and without ASD. Five potential candidates come to mind that may moderate planning ability in ASD. First, severity of ASD symptoms may affect planning performance. Across studies included in this meta-analysis, all DSM-IV-TR (APA 2000) subtypes of ASD (Asperger, PDD-NOS or autism) were assigned to one overall ASD category (in line with DSM-5; APA 2013) due to missing specification of this information within the studies. However, some previous reports suggest that EF difficulties increase as symptoms of ASD are more severe. For example, Bölte et al. (2011) found that higher planning difficulties were associated with higher scores for stereotypic, ritualized behavior and interests on the ADI-R and ADOS. These symptoms thus may specify the extent of planning difficulties, and, therefore, ASD symptomatology might even be more interesting to investigate as a moderator of planning ability. Unfortunately, we were unable to do this in the current study, as information on ASD symptomatology was not sufficiently reported in the included studies and the studies that did report on ASD symptoms used a variety of measures, which complicates a moderator analysis. We, therefore recommend that in future studies the relationship between ASD symptomatology and planning ability in people with ASD will be tested.

Second, comorbid psychopathology may influence performance on lab-based planning measures in people with ASD. People with ASD have higher rates of psychiatric comorbidity than people without ASD; 69% percent of people with ASD as opposed to 40% of typically developing people meet criteria for another psychiatric disorder at least once in their life (Buck et al. 2014; Kessler et al. 2005). Psychiatric disorders other than ASD are also related to poorer cognitive functioning (e.g. McDermot and Ebmeier 2009), and thus, it may be that the higher incidence of psychiatric comorbidity in ASD partly explains why planning is worse in those with ASD as compared to people without ASD. Further study is needed to determine the potential impact of comorbid psychiatric disorders on planning performance in people with ASD.

Third, the use of psychotropic medication may affect planning performance in people with ASD. It is well-known that the majority of people with ASD use some type of psychotropic medication (Esbensen et al. 2009), especially those with comorbid psychiatric disorders (Coury et al. 2012), and that the use of this medication can have adverse effects on cognitive performance (Agay et al. 2010; Amado-Boccara et al. 1995; Linssen et al. 2014). We, therefore, recommend including measures of these factors in future studies on planning performance in people with ASD in order to further explain the heterogeneity across planning studies.

Fourth, related to the impact of IQ, mental age might also be an informative factor for moderation as two individuals with the same IQ may be functioning on different developmental levels (i.e., have a different mental age). Mental age might, therefore, capture the individuals’ level of intellectual functioning better than IQ tasks. However, we could not explore the impact of mental age on the variability in effect sizes in our meta-analysis as only five studies reported on mental age. This number is insufficient to make any valid statements concerning moderation of planning deficits in ASD by mental age, but should definitely be investigated in future studies.

Fifth, the choice of comparison group may impact meta-analytic findings. For example, using a comparison group of unaffected siblings of individuals with ASD or a specific clinical group will lead to different, possible smaller, effect sizes, than using a TD comparison group. However, as only one of the included studies had unaffected siblings as the comparison group (Bölte et al. 2011), it is unlikely that this affected our results.

An additional important avenue of future research that cannot be covered within a meta-analysis, is the investigation of individual differences in planning ability.^4^ Previous studies show that individual differences in planning ability exist in both people with and without ASD (e.g. Brunsdon et al. 2015; Hill and Bird 2006; Hughes et al. 1994; Wallace et al. 2016). Focusing on individual differences instead of group comparisons can help to determine whether specific subgroups within the ASD group exist with respect to planning performance.

Despite the limitations in relation to the unexplained heterogeneity and the publication bias, the observed planning difficulties of people with ASD as compared to typically developing individuals underline that there might be room for improvement with respect to the planning abilities in people with ASD. As planning is so key to our daily life, intervention aimed at improving this skill might be helpful for people with ASD. The meta-analysis further suggests that it is of importance that null findings (and counter intuitive findings) need to be published as only then we can determine which of the currently studied factors (i.e., moderators) influencing planning abilities can indeed be fully dismissed. Nonetheless, we also need to investigate additional factors that could explain heterogeneity in effects to help unravel the planning deficit among individuals with ASD.

## Footnotes

1 Note that the EMBASE and CINAHL databases were also considered for the systematic literature search, but not included as they largely overlap with the PubMed database and because their added value in comparison to PubMed, namely more coverage of respectively pharmacological and nursing research, was not of specific interest given our topic of interest. Therefore, we chose to use PubMed rather than EMBASE or CINAHL.

2 Note that we chose to not include studies using the Trail Making Test (Reitan and Wolfson 1985) which was reported on in the last qualitative review of Hill (2004). Rather than a pure measure of planning, it assesses a number of different functions related to mental flexibility (Crowe 1998; Delis et al. 2001). In addition, tasks were not included if they did not assess the cognitive ability of thinking ahead, such as motor planning tasks, or tasks that were not commonly known in the planning literature and of which we, therefore, did not know whether they validly assessed planning. For example, one of the tasks that we did not include was the Question Discrimination and Plan Construction task used in Alderson-Day (2011), as this task was not used in any other ASD planning study and is not widespread in the planning literature.

3 A rerun of our meta-analysis in which we set the inter-test correlation to *r* = .41 for the studies of which the inter-test correlation was unknown gave the same main outcome of a significant medium positive effect size of 0.52.

4 Please note that heterogeneity on the individual level (for example, variability within one group on planning task) is a different type of heterogeneity than the examined heterogeneity in the current meta-analysis (variability in study findings on group differences on planning task).

## References

[CR1] Agay N, Yechiam E, Carmel Z, Levkovitz Y (2010). Non-specific effects of methylphenidate (Ritalin) on cognitive ability and decision-making of ADHD and healthy adults. Psychopharmacology.

[CR2] Alderson-Day B (2011). Verbal problem-solving in autism spectrum disorders: A problem of plan construction?. Autism Research.

[CR3] Amado-Boccara I, Gougoulis N, Littre MP, Galinowski A, Loo H (1995). Effects of antidepressants on cognitive functions: a review. Neuroscience & Biobehavioral Reviews.

[CR4] American Psychiatric Association (2000). Diagnostic and Statistical Manual of Mental. Disorders.

[CR5] American Psychiatric Association (2013). Diagnostic and Statistical Manual of Mental Disorders.

[CR6] Anderson VA, Anderson P, Northam E, Jacobs R, Catroppa C (2001). Development of executive functions through late childhood and adolescence in an Australian sample. Developmental Neuropsychology.

[CR7] Best JR, Miller PH, Jones LL (2009). Executive functions after age 5: Changes and correlates. Developmental Review.

[CR8] Bölte S, Duketis E, Poustka F, Holtmann M (2011). Sex differences in cognitive domains and their clinical correlates in higher-functioning autism spectrum disorders. Autism.

[CR9] Booth R, Charlton R, Hughes C, Happé F (2003). Disentangling weak coherence and executive dysfunction: planning drawing in autism and attention–deficit/hyperactivity disorder. Philosophical Transactions of the Royal Society B.

[CR10] Borenstein M, Hedges LV, Higgins JPT, Rothstein HR (2009). Introduction to meta-analysis.

[CR11] Boucher J, Cowell P, Howard M, Broks P, Farrant A, Roberts N, Mayes A (2005). A combined clinical, neuropsychological, and neuroanatomical study of adults with high functioning autism. Cognitive Neuropsychiatry.

[CR12] Bramham J, Ambery F, Young S, Morris R, Russell A, Xenitidis K, Murphy D (2009). Executive functioning differences between adults with attention deficit hyperactivity disorder and autistic spectrum disorder in initiation, planning and strategy formation. Autism.

[CR13] Brunsdon VEA, Colvert E, Ames C, Garnett T, Gillan N, Hallet V, Happé F (2015). Exploring the cognitive features in children with autism spectrum disorder, their co-twins, and typically developing children within a population-based sample. Journal of Child Psychology and Psychiatry.

[CR14] Buck TR, Viskochil J, Farley M, Coon H, McMahon WM, Morgan J, Bilder DA (2014). Psychiatric comorbidity and medication use in adults with autism spectrum disorder. Journal of Autism and Developmental Disorders.

[CR15] Burgess PW, Simons JS, Dumontheil I, Gilbert SJ, Duncan J, Philips L, McLeod P (2005). The gateway hypothesis of rostral prefrontal cortex (area 10) function. Measuring the mind: Speed, control, and age.

[CR16] Corbett BA, Constantine LJ, Hendren R, Rocke D, Ozonoff S (2009). Examining executive functioning in children with autism spectrum disorder, attention deficit hyperactivity disorder and typical development. Psychiatry Research.

[CR17] Coury DL, Anagnostou E, Manning-Courtney P, Reynolds A, Cole L, McCoy R, Perrin JM (2012). Use of psychotropic medication in children and adolescents with autism spectrum disorders. Pediatrics.

[CR18] Crowe SF (1998). The differential contribution of mental tracking, cognitive flexibility, visual search, and motor speed to performance on parts A and B of the Trail Making Test. Journal of Clinical Psychology.

[CR19] Dang C-P, Braeken J, Colom R, Ferrer E, Liu C (2014). Why is working memory related to intelligence? Different contributions from storage and processing. Memory (Hove, England).

[CR20] Delis DC, Kaplan E, Kramer JH (2001). The Delis-Kaplan executive function system: Examiner’s manual.

[CR21] Duval S, Tweedie R (2000). Trim and Fill: A simple funnel-plot-based method for testing and adjusting for publication bias in meta-analysis. Biometrics.

[CR22] Esbensen AJ, Greenberg JS, Seltzer MM, Aman MG (2009). A longitudinal investigation of psychotropic and non-psychotropic medication use among adolescents and adults with autism spectrum disorders. Journal of autism and developmental disorders.

[CR23] Friedman NP, Miyake A, Corley RP, Young SE, DeFries JC, Hewitt JK (2006). Not all executive functions are related to intelligence. Psychological Science.

[CR25] Geurts HM, Verté S, Oosterlaan J, Roeyers H, Sergeant JA (2004). How specific are executive functioning deficits in attention deficit hyperactivity disorder and autism?. Journal of Child Psychology and Psychiatry.

[CR26] Geurts HM, Vissers ME (2012). Elderly with autism: Executive functions and memory. Journal of Autism and Developmental Disorders.

[CR27] Goldberg MC, Mostofsky SH, Cutting LE, Mahone EM, Astor BC, Denckla MB, Landa RJ (2005). Subtle executive impairment in children with autism and children with ADHD. Journal of Autism and Developmental Disorders.

[CR28] Griebling J, Minshew NJ, Bodner K, Libove R, Bansal R, Konasale P, Hardan A (2010). Dorsolateral prefrontal cortex magnetic resonance imaging measurements and cognitive performance in autism. Journal of Child Neurology.

[CR29] Hanson LK, Atance CM (2014). Brief report: episodic foresight in autism spectrum disorder. Journal of Autism and Developmental Disorders.

[CR30] Happé F, Booth R, Charlton R, Hughes C (2006). Executive function deficits in autism spectrum disorders and attention-deficit/hyperactivity disorder: Examining profiles across domains and ages. Brain and Cognition.

[CR31] Hedges LV, Olkin I (1985). Statistical models for meta-analysis.

[CR32] Hill EL (2004). Executive dysfunction in autism. Trends in Cognitive Sciences.

[CR33] Hill EL, Bird CM (2006). Executive processes in Asperger syndrome: Patterns of performance in multiple case series. Neuropsychologia.

[CR34] Hughes C, Russell J, Robbins TW (1994). Evidence for executive dysfunction in autism. Neuropsychologia.

[CR35] Huizinga M, Dolan CV, van der Molen MW (2006). Age-related change in executive function: Developmental trends and a latent variable analysis. Neuropsychologia.

[CR36] Huizinga M, Smidts DP (2011). Age-related changes in executive function: A normative study with the Dutch version of the Behavior Rating Inventory of Executive Function (BRIEF). Child Neuropsychology.

[CR37] Joseph RM, McGrath LM, Tager-Flusberg H (2005). Executive dysfunction and its relation to language ability in verbal school-age children with autism. Developmental Neuropsychology.

[CR38] Just MA, Cherkassky VL, Keller TA, Kana RK, Minshew NJ (2007). Functional and anatomical cortical underconnectivity in autism: Evidence from an FMRI study of an executive function task and corpus callosum morphometry. Cerebral Cortex.

[CR39] Kaufmann L, Zotter S, Pixner S, Starke M, Haberlandt E, Steinmayr-Gensluckner M, Marksteiner J (2013). Brief report: CANTAB performance and brain structure in pediatric patients with Asperger syndrome. Journal of Autism and Developmental Disorders.

[CR40] Keary CJ, Minshew NJ, Bansal R, Goradia D, Fedorov S, Keshavan MS, Hardan AY (2009). Corpus callosum volume and neurocognition in autism. Journal of Autism and Developmental Disorders.

[CR41] Kenworthy L, Yerys BE, Anthony LG, Wallace GL (2008). Understanding executive control in autism spectrum disorders in the lab and in the real world. Neuropsychology review.

[CR42] Kessler RC, Berglund P, Demler O, Jin R, Merikangas KR, Walters EE (2005). Lifetime prevalence and age-of-onset distributions of DSM-IV disorders in the national comorbidity survey replication. Archives of General Psychiatry.

[CR43] Kimhi Y, Shoam-Kugelmas D, Ben-Artzi GA, Ben-Moshe I, Bauminger-Zviely N (2014). Theory of mind and executive function in preschoolers with typical development versus intellectually able preschoolers with autism spectrum disorder. Journal of Autism and Developmental Disorders.

[CR44] Landa RJ, Goldberg MC (2005). Language, social, and executive functions in high functioning autism: A continuum of performance. Journal of Autism and Developmental Disorders.

[CR46] Limoges É, Bolduc C, Berthiaume C, Mottron L, Godbout R (2013). Relationship between poor sleep and daytime cognitive performance in young adults with autism. Research in Developmental Disabilities.

[CR47] Lin CS, Chang SH, Liou WY, Tsai YS (2013). The development of a multimedia online language assessment tool for young children with autism. Research in Developmental Disabilities.

[CR48] Linssen AMW, Sambeth A, Vuurman EFPM, Riedel WJ (2014). Cognitive effects of methylphenidate in healthy volunteers: A review of single dose studies. International Journal of Neuropsychopharmacology.

[CR49] Lopez BR, Lincoln AJ, Ozonoff S, Lai Z (2005). Examining the relationship between executive functions and restricted, repetitive symptoms of autistic disorder. Journal of Autism and Developmental Disorders.

[CR50] Losh M, Adolphs R, Poe MD, Couture S, Penn D, Baranek GT, Piven J (2009). Neuropsychological profile of autism and the broad autism phenotype. Archives of General Psychiatry.

[CR51] Low J, Goddard E, Melser J (2009). Generativity and imagination in autism spectrum disorder: Evidence from individual differences in children’s impossible entity drawings. British Journal of Developmental Psychology.

[CR52] Luna B, Doll SK, Hegedus SJ, Minshew NJ, Sweeney JA (2007). Maturation of executive function in autism. Biological Psychiatry.

[CR53] Matson JL, Horovitz M (2010). Stability of autism spectrum disorders symptoms over time. Journal of Developmental and Physical Disabilities.

[CR54] McCrimmon AW, Schwean VL, Saklofske DH, Montgomery JM, Brady DI (2012). Executive functions in Asperger’s syndrome: An empirical investigation of verbal and nonverbal skills. Research in Autism Spectrum Disorders.

[CR55] McDermott LM, Ebmeier KP (2009). A meta-analysis of depression severity and cognitive function. Journal of Affective Disorders.

[CR56] McGovern CW, Sigman M (2005). Continuity and change from early childhood to adolescence in autism. Journal of Child Psychology and Psychiatry.

[CR57] McLean RL, Harrison AJ, Zimak E, Joseph RM, Morrow EM (2014). Executive function in probands with autism with average IQ and their unaffected first-degree relatives. Journal of the American Academy of Child and Adolescent Psychiatry.

[CR58] Medeiros K, Winsler A (2014). Parent–child gesture use during problem solving in autistic spectrum disorder. Journal of Autism and Developmental Disorders.

[CR59] Mesulam M, Stuss DT, Knight RT (2002). The human frontal lobes: Transcending default mode through contingent encoding. Principles of frontal lobe function.

[CR60] Moher, D., Shamseer, L., Clarke, M., Ghersi, D., Liberati, A., Petticrew, M., Stewart, L. A. (2015). Preferred reporting items for systematic review and meta-analysis protocols (PRISMA-P) 2015 statement. *Systematic Reviews*, 4(1). doi:10.1186/2046-4053-4-1. 10.1186/2046-4053-4-1 PMC4320440 25554246

[CR61] O’Hearn K, Asato M, Ordaz S, Luna B (2008). Neurodevelopment and executive function in autism. Development and Psychopathology.

[CR62] Olivar-Parra JS, De-La-Iglesia-Gutiérrez M, Forns M (2011). Training referential communicative skills to individuals with autism spectrum disorder: A pilot study. Psychological Reports.

[CR63] Ozonoff S, Cook I, Coon H, Dawson G, Joseph RM, Klin A, Wrathall D (2004). Performance on Cambridge Neuropsychological Test Automated Battery subtests sensitive to frontal lobe function in people with autistic disorder: Evidence from the collaborative programs of excellence in autism network. Journal of Autism and Developmental Disorders.

[CR64] Ozonoff S, Dawson G, McPartland J (2002). A parent’s guide to asperger syndrome and high-functioning autism: How to meet the challenges and help your child thrive.

[CR65] Ozonoff S, Jensen J (1999). Brief report: Specific executive function profiles in three neurodevelopmental disorders. Journal of Autism and Developmental Disorders.

[CR66] Ozonoff S, McEvoy RE (1994). A longitudinal study of executive function and theory of mind development in autism. Development and Psychopathology.

[CR67] Panerai S, Tasca D, Ferri R, Genitori D’Arrigo V, Elia M (2014). Executive functions and adaptive behaviour in autism spectrum disorders with and without intellectual disability. Psychiatry Journal.

[CR68] Pellicano E (2007). Links between theory of mind and executive function in young children with autism: Clues to developmental primacy. Developmental Psychology.

[CR69] Pellicano E (2010). The development of core cognitive skills in autism: A 3-year prospective study. Child Development.

[CR70] Pellicano E, Maybery M, Durkin K, Maley A (2006). Multiple cognitive capabilities/deficits in children with an autism spectrum disorder: “Weak” central coherence and its relationship to theory of mind and executive control. Development and Psychopathology.

[CR71] Planche P, Lemonnier E (2012). Children with high-functioning autism and Asperger’s syndrome: Can we differentiate their cognitive profiles?. Research in Autism Spectrum Disorders.

[CR72] Prior M, Hoffmann W (1990). Brief report: Neuropsychological testing of autistic children through an exploration with frontal lobe tests. Journal of Autism and Developmental Disorders.

[CR73] Rajendran G, Law AS, Logie RH, Van Der Meulen M, Fraser D, Corley M (2011). Investigating multitasking in high-functioning adolescents with autism spectrum disorders using the Virtual Errands Task. Journal of Autism and Developmental Disorders.

[CR74] Rajendran G, Mitchell P, Rickards H (2005). How do individuals with Asperger syndrome respond to nonliteral language and inappropriate requests in computer-mediated communication?. Journal of Autism and Developmental Disorders.

[CR75] Reitan RM, Wolfson D (1985). The Halstead–Reitan neuropsycholgical test battery.

[CR76] Robinson S, Goddard L, Dritschel B, Wisley M, Howlin P (2009). Executive functions in children with autism spectrum disorders. Brain and Cognition.

[CR77] Romine C, Reynolds C (2005). A model of the development of frontal lobe functioning: Findings from a meta-analysis. Applied Neuropsychology.

[CR78] Rosenthal M, Wallace GL, Lawson R, Wills MC, Dixon E, Yerys BE, Kenworthy L (2013). Impairments in real-world executive function increase from childhood to adolescence in autism spectrum disorders. Neuropsychology.

[CR79] Rosenthal R (1979). The “file drawer problem” and tolerance for null results. Psychological Bulletin.

[CR80] Ruta L, Mugno D, D’Arrigo VG, Vitiello B, Mazzone L (2010). Obsessive–compulsive traits in children and adolescents with Asperger syndrome. European Child and Adolescent Psychiatry.

[CR81] Rutter M, Rutter M, Schopler E (1978). Diagnosis and definition. Autism: A reappraisal of concepts of treatment.

[CR82] Sachse M, Schlitt S, Hainz D, Ciaramidaro A, Schirman S, Walter H, Freitag CM (2013). Executive and visuo-motor function in adolescents and adults with autism spectrum disorder. Journal of Autism and Developmental Disorders.

[CR83] Schurink J, Hartman E, Scherder EJA, Houwen S, Visscher C (2012). Relationship between motor and executive functioning in school-age children with pervasive developmental disorder not otherwise specified. Research in Autism Spectrum Disorders.

[CR84] Semrud-Clikeman M, Walkowiak J, Wilkinson A, Butcher B (2010). Executive functioning in children with Asperger syndrome, ADHD-combined type, ADHD-predominately inattentive type, and controls. Journal of Autism and Developmental Disorders.

[CR85] Sergeant JA, Geurts H, Oosterlaan J (2002). How specific is a deficit of executive functioning for attention-deficit/hyperactivity disorder?. Behavioural Brain Research.

[CR86] Sinzig J, Morsch D, Bruning N, Schmidt MH, Lehmkuhl G (2008). Inhibition, flexibility, working memory and planning in autism spectrum disorders with and without comorbid ADHD-symptoms. Child and Adolescent Psychiatry and Mental Health.

[CR87] Taddei S, Contena B (2013). Brief Report: Cognitive performance in autism and asperger’s syndrome: What are the differences?. Journal of Autism and Developmental Disorders.

[CR88] Turner HM, Bernard RM (2006). Calculating and synthesizing effect sizes. Contemporary Issues in Communication Science and Disorders.

[CR89] Unterrainer JM, Rauh R, Rahm B, Hardt J, Kaller CP, Klein C, Paschke-Müller M, Biscaldi M (2015). Development of planning in children with high-functioning autism spectrum disorders and/or attention deficit/hyperactivity disorder. Autism Research.

[CR90] Van den Bergh SF, Scheeren AM, Begeer S, Koot HM, Geurts HM (2014). Age related differences of executive functioning problems in everyday life of children and adolescents in the autism spectrum. Journal of Autism and Developmental Disorders.

[CR91] Van Eylen L, Boets B, Steyaert J, Wagemans J, Noens I (2015). Executive functioning in autism spectrum disorders: Influence of task and sample characteristics and relation to symptom severity. European Child & Adolescent Psychiatry.

[CR92] Verté S, Geurts HM, Roeyers H, Oosterlaan J, Sergeant JA (2005). Executive functioning in children with autism and Tourette syndrome. Development and Psychopathology.

[CR93] Verté S, Geurts HM, Roeyers H, Oosterlaan J, Sergeant JA (2006). Executive functioning in children with an autism spectrum disorder: Can we differentiate within the spectrum?. Journal of Autism and Developmental Disorders.

[CR94] Viechtbauer W (2010). Conducting meta-analyses in R with the metaphor package. Journal of Statistical Software.

[CR95] Wallace GL, Kenworthy L, Pugliese CE, Popal HS, White EI, Brodsky E, Martin A (2016). Real-world executive functions in adults with autism spectrum disorder: Profiles of impairment and associations with adaptive functioning and co-morbid anxiety and depression. Journal of autism and developmental disorders.

[CR96] Wallace GL, Silvers JA, Martin A, Kenworthy LE (2009). Brief report: Further evidence for inner speech deficits in autism spectrum disorders. Journal of Autism and Developmental Disorders.

[CR97] Ward G, Morris R, Morris R, Ward G (2005). Introduction to the psychology of planning. The Cognitive Psychology of Planning.

[CR98] White SJ, Burgess PW, Hill EL (2009). Impairments on “open-ended” executive function tests in autism. Autism Research.

[CR99] Williams D, Jarrold C (2013). Assessing planning and set-shifting abilities in autism: Are experimenter-administered and computerised versions of tasks equivalent?. Autism Research.

[CR100] Williams DL, Mazefsky CA, Walker JD, Minshew NJ, Goldstein G (2014). Associations between conceptual reasoning, problem solving, and adaptive ability in high-functioning autism. Journal of Autism and Developmental Disorders.

[CR101] Williams DM, Bowler DM, Jarrold C (2012). Inner speech is used to mediate short-term memory, but not planning, among intellectually high-functioning adults with autism spectrum disorder. Development and Psychopathology.

[CR102] Zinke K, Fries E, Altgassen M, Kirschbaum C, Dettenborn L, Kliegel M (2010). Visuospatial short-term memory explains deficits in tower task planning in high-functioning children with autism spectrum disorder. Child Neuropsychology.

